# Pasture Characteristics in Three Different Ecotypes at Khovd Aimag, Western Mongolia

**DOI:** 10.1371/journal.pone.0102892

**Published:** 2014-07-24

**Authors:** Jutta Beher

**Affiliations:** ARC Centre of Excellence for Environmental Decisions, , University of Queensland, Brisbane, Queensland, Australia; National University of Mongolia, Mongolia

## Abstract

The transition of nomadic pastoralism to more sessile forms of rangeland utilization and increased stocking rates can result in the degradation of pasture. After political changes in the 1990s in Mongolia, population growth and missing alternative livelihoods intensified the grazing pressure on pastures, and further decreased the condition of the fragile arid ecosystems. To learn more about the productivity and quality of pasture land in Khovd Aimag in the western region of Mongolia, standing biomass was measured in the alpine region, mountain steppe and semi-desert. Plant samples were analyzed for nitrogen and fiber contents by wet chemistry and Near Infrared Spectroscopy (NIRS). Results show clear differences in distribution of biomass with reduced biomass in the vicinity of temporary settlements. From July to early September plant nitrogen contents decreased in the alpine region, remained unchanged in the mountain steppe and increased in the semi-desert. Nitrogen concentrations were elevated in vegetation close to temporary settlements. For fiber contents (ADF) no clear patterns were found. Neither biomass/m^2^ nor vegetation cover were appropriate indicators for food quality.

## Introduction

Almost half of the Mongolian human population depends on livestock in a direct or indirect way. About 75% of the vast country is used as pasture for goats, sheep, cattle, horses and camels [1]. For hundreds of years pastoralists pursued a semi-nomadic lifestyle, moving livestock and households among seasonal pastures about four times per year to meet the nutritional demands of their animals, and to conserve pasture resources [2]–[4]. Climate on the Mongolian Plateau has been getting warmer and drier since the early 1960s [5], [6]. The number of droughts and winter snowstorms has increased in Mongolia over the last 50 years, particularly in the last decade [7]. Due to lack of effective resource institutions, conflicts among herders over grassland use have increased since the early 1990s [3] and degradation of many areas has become an issue due to socio-economic changes and increased stocking rates [2], [6]. At the same time, distance and frequency of nomadic movement have declined [8]. The transition from ancient tradition to centralized organization and back to individually organized rangeland organization, for example in parts of Russia and China, has led to severe degradation of pasture ecosystems in the recent past. The same is now probable in rural Mongolia, and coherent management plans still do not exist. The growth of the human population of Mongolia requires intensified productivity in the agricultural sector, which increases the pressure on pastures [4], [9]–[11]. Still, relatively little is known about most Mongolian steppe ecosystems and their dynamics. Opinions on correct classification of underlying general dynamics of Mongolian rangelands differ immensely [12]–[15]. For sustainable livestock management, data of seasonal and site specific pasture conditions and response to climate and grazing are needed [12]. The suitability of an area depends not only on food biomass but also on food quality. Most ongoing research focuses on vegetation change and plant species but little is known about the nutritional quality of the pasture systems in time and space.

Apart from quantitative differences in food availability, the chemical composition of plants changes during the growing season. Besides the secondary plant chemicals (most important in herbs and woody plants), food quality for livestock is characterized by nitrogen and fiber concentrations with the ratio of nitrogen-to-fiber used as a measure of food quality for ruminants [16]. In general, concentrations of nitrogen decrease while fibers increase as the plants grow older [17]–[22].

In order to come to a better understanding of the spatial and temporal pattern of food for the livestock of nomadic herdsmen in Mongolia, I analyzed mixed vegetation samples from pastures in three different ecotypes. I addressed the following questions: (1) What are the quantitative and qualitative changes of vegetation during growing season? (2) How does the quantity and quality of the vegetation differ between temporary inhabited valleys and areas further away from these settlements?

## Materials and Methods

### Study area

**Figure 1:**
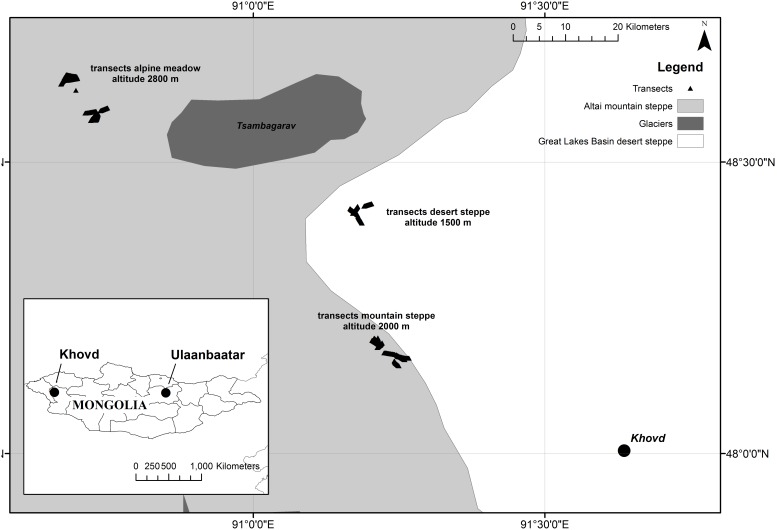
Study area in the northwest of Khovd City. Study sites at desert steppe in the area of the Great Lakes Basin, alpine region close to mount Tsambagarav and mountain steppe.

The study was carried out in the north-west of Khovd city in Khovd Aimag in Western Mongolia (48°01′N, 91°34′E) (Figure 1).

The area extends across three different ecotypes from the semi-deserts of the Great Lake Basin to mountain steppe and alpine regions in the Altai Mountains [23], [24]. These ecotypes are three major ecological zones in Mongolia and their close vicinity at the Khovd region simplifies comparative studies. The distance between the studied areas was about 30–80 kilometers (Figure 1).

**Figure 2:**
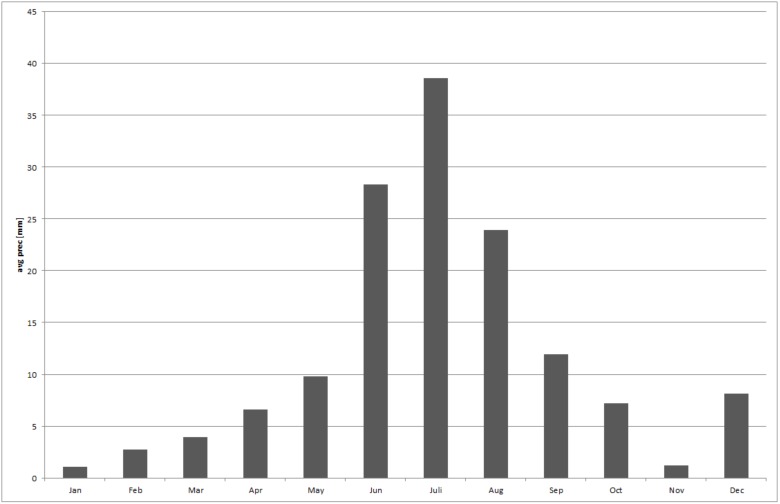
Mean amount of rainfall at Khovd Region from 1983 to 2004 [47].

Annual rainfall in Mongolia ranges from 50 mm/year in the southern regions and 500 mm/year in the northern mountains. Up to 96% of the rains fall in summer. Data from the weather station at Khovd (1405 m above sea level) show a clear peak between May and September (Figure 2).

The time of increased precipitation represents the general growth period of vegetation, however, the time span of climate conditions that allow growth of plants is much shorter in higher regions, with a later start and an earlier ending than in lower regions.

Extreme temperatures, drought and short transitions between seasons result in limited soil moisture. Therefore, soil formation occurs only in thin layers [25], and strong winds can denude the sparsely covered soils. The alpine region and upper mountain steppe show kastanozems, semi-desert regions mostly burozems. The alpine region above 2700 m altitude is dominated by sedgegrass and kobresia meadows, mountain steppe (1800–2500 m) and semi-deserts (1200–1700 m) by scant graminoid communities dominated by *Stipa* sp. and often assorted with dwarf bushes like *Caragana* sp. The whole area is used for pasture by nomad families and their animals who settle for short spans of time, averaging several weeks, at different riverbanks. Gers are set up on their arrival and deconstructed again shortly before the next migration. This way, temporary villages of 20–50 gers are built, demolished and rebuilt over time and place. Rivers are used as a watersource for human beings and animals. The use of wells was not observed in the studied area. Most people own the five common livestock species of yaks, camels, horses, sheep and goats. The former three are low in numbers and graze on their own, while sheep and goats dominate in numbers and are kept in mixed herds which are driven by a herdsman with daily path-lengths of about 10 km. Every family in the study area owns at least sheep and goats in a mixed herd of a minimum of 100 animals. Most families follow their own schedule coming back to the same places for many years. Nomads in the study area used the alpine region and mountain steppe for pasture from July 15 to August 20, and semi-desert from August 13 until September 2 [26].

### Sampling design

Biomass and nutrient dynamics were studied in relation to: (1) seasonality, (2) ecotype and (3) distance to a river in valleys inhabited temporarily during the summer months.

Seasonality is defined as time span of active plant physiology and was assessed by sampling in July, August and September, which are the main months of the growth period. Ecotype is defined as semi-desert (sampling site at 1700 m, 48°25′N, 91°10′E), mountain steppe (sampling site at 2000 m, 48°11′N, 91°12′E and 48°9′N, 91°15′E) and alpine region (sampling site at 2700 m, 48°38′N, 90°40′E and 48°34′N, 90°43′E). Distance to a river implies two sets of plots within an ecotype differing in distance to the next temporarily inhabited riverbank. Ger numbers at the study sites were about 50 at alpine region and mountain steppe and 25 at semi-desert.

Distance to the river with the associated settlements was assumed to indicate utilization intensity by livestock. This was verified by tracking of herds with GPS collars: A range of 2 km on both sides of the rivers seemed to be used more frequently by livestock than areas outside this zone [26]. Distance classes are referred to as “distant” and “close”. Close plots have less than 2 km distance to the riverbank, distant ones are more remote but not more than 5 km away.

**Figure 3:**
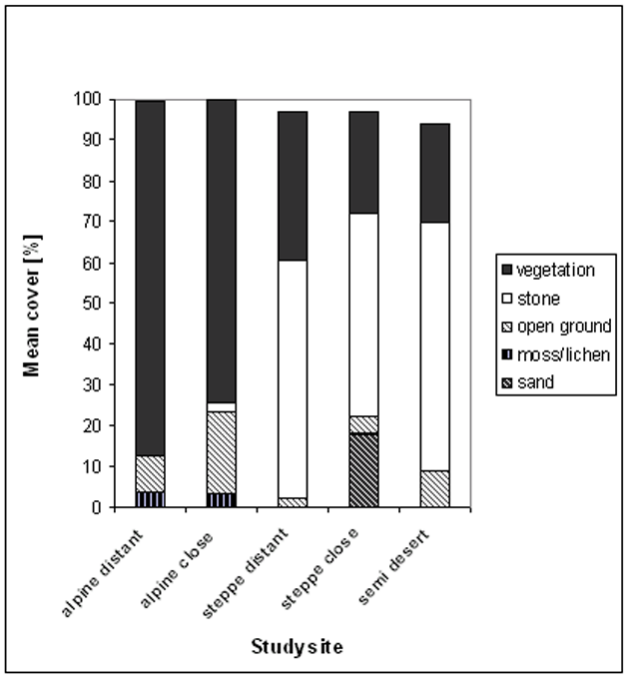
Mean cover of vegetation and particle size of the ground at study sites of alpine region, mountain steppe and semi-desert.

Arrangement and distance between plots should create a representative sample for the studied pasture. To avoid geomorphological or topographical effects [15], [27], large homogenous areas were chosen that had no difference in slope or aspect and were typical examples of the ecotype [25]. The areas directly adjacent to a river were classified as a different ecotype and were not sampled. In the mountain steppe and the alpine region one transect of 1.5 km length was established in a random direction at the two distance classes each month. Starting from a random point and using a GPS device for bearing, every 75 m a 1 m^2^ plot was sampled, resulting in 20 different plots along each transect (Table 1) Exposure and slope where consistent for all three transects within each ecotype. For each 1 m^2^ plot the vegetation composition was described on the species level following Braun-Blanquet [28]. Vegetation descriptions included all herbaceous plants, graminoids, and shrubs to fully describe the ecotype and its plant communities (Table S1 “Species List”). Shrub species were excluded in the biochemical analysis to avoid bias, because the probability that they would frequently occur in the plots along transects was low. In the rare occasion that a caragana bush occurred inside of a plot, the plot was established 1 m behind the bush to avoid effects of shading or root competition. The percentage cover of moss/lichen, stone, sand, bare soil and vegetation were estimated. Stone, sand, and bare soil were classified according to particle size: stone>2 mm, sand 0.1–2 mm and bare soil<0.1 mm (Figure 3).

**Table 1:** Sampling design: 20 plots of 1 m^2^ were sampled per month and distance class in the alpine region and the mountain steppe; and 20 plots were sampled per month in the semi-desert.

	Alpine region	Mountain steppe	Semi-desert
distant	July: 1 transect with 20 plots	July: 1 transect with 20 plots	July: 1 transect with 20 plots
	Aug: 1 transect with 20 plots	Aug: 1 transect with 20 plots	Aug: 1 transect with 20 plots
	Sept: 1 transect with 20 plots	Sept: 1 transect with 20 plots	
close	July: 1 transect with 20 plots	July: 1 transect with 20 plots	
	Aug: 1 transect with 20 plots	Aug: 1 transect with 20 plots	
	Sept: 1 transect with 20 plots	Sept: 1 transect with 20 plots	Sept: 1 transect with 20 plots

At the semi-desert only one transect was established per month. Due to the late arrival of nomad families it was unknown where they would install their camps at the riverbank. Therefore, classification of close and distant sites was possible only afterwards and data for the semi-desert is incomplete. This data is included in correlations but not in the multivariate analysis.

### Plant sampling for biomass determination and chemical analyses

Plant material was collected every third/fourth week from the beginning of July through to early September. All plant material was cut at 1 cm height above ground in each of the twenty 1 m^2^ plots per transect. The field work was conducted in collaboration with the University of Khovd and no additional specific permission was required for any sampling at the study sites. The accessed land was neither privately owned nor protected and no endangered or protected species were involved in the sampling. All locations were subject to temporary livestock grazing. In total 300 plots were sampled (Table 1).

Biomass was defined as above-ground Dry Organic Matter (DOM [g/m^2^]) measured after 24 h of drying at 60°C in an oven. Food quality was defined as the ratio of Acid Detergent Fiber and nitrogen content (N/ADF).

Of the 300 samples of 1 m^2^ plots 291 plant samples were analyzed for total nitrogen content using Kjeldahl digestion with sulphuric acid at 400°C and Near-Infra-Red-Spectroscopy (NIRS) [29]–[31]. In addition, fiber contents were measured as acid detergent fiber (ADF) extraction according to Van Soest et al. [18].

Relationships between nitrogen, fiber, quality and biomass using ecotype, season and distance class as independent variables were analyzed with ANOVA. Data were log-transformed prior to analysis to meet ANOVA assumptions. Relationships between biomass and chemical characteristics were checked with simple correlations. Statistics are based on the values of the single plots.

## Results

### Ecotype

**Figure 4:**
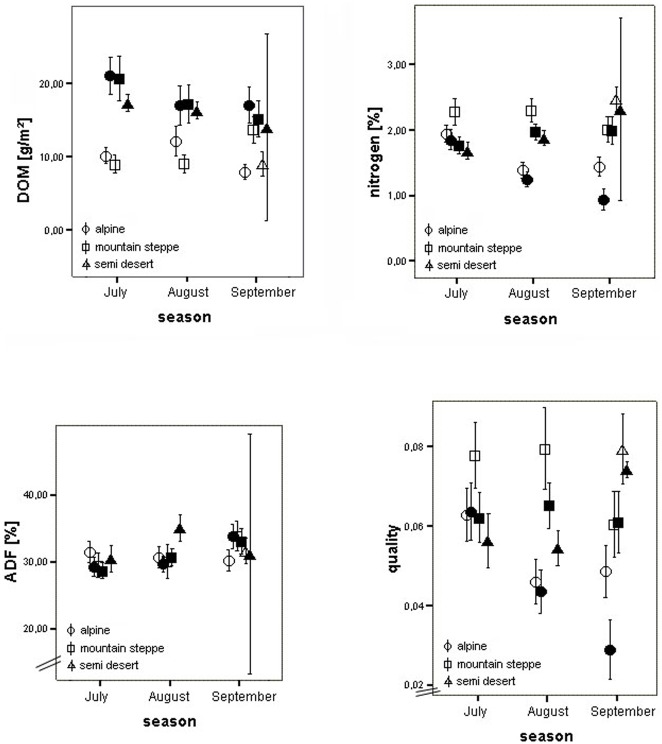
DOM, nitrogen, ADF and quality (N/ADF) of vegetation of 1 m^2^ plots in relation to ecotype, season and distance class of study sites. Error bars indicate the 95% confidence interval of the mean. Empty symbols = sites close to rivers, filled symbols = sites away from rivers. Data for the semi-desert were not assigned to close or distant sites.

The number of plant species is highest in the mountain steppe and lowest in the semi-desert (Table S1 “Species List”). Biomass does not differ between ecotypes although vegetation cover is highly different across ecotypes. Alpine meadows show a very high cover in comparison to the sparse grass communities of semi-desert and mountain steppe. Altitudinal differences were primarily found for nitrogen and quality, with lower values in alpine region (Table 2, Figure 4).

**Table 2:** Effects of the ecotype, seasonality and distance to the river on the biomass, nitrogen, ADF and food quality in the alpine meadow and the mountain steppe according to ANOVA.

Factor	df	Biomass		Nitrogen		ADF		Quality	
		F	P	F	p	F	p	F	p
Ecotype	1	0.1622	0.686	**239.8722**	<0.0001	0.0796	0.778	**127.1525**	<0.0001
Seasonality	2	1.9426	0.146	**38.6886**	<0.0001	**14.8750**	<0.0001	**37.2654**	<0.0001
Distance	1	**196.7121**	<0.0001	**52.1571**	<0.0001	0.0027	0.959	**27.1747**	<0.0001
Ecotype* Seasonality	2	**6.6454**	0.002	**40.6727**	<0.0001	**3.0549**	0.049	**15.6000**	<0.0001
Ecotype* Distance	1	0.5521	0.459	1.5799	0.210	0.1462	0.702	1.2844	0.25834
Seasonality * Distance	2	**5.7054**	0.004	2.1194	0.123	**3.5532**	0.030	**3.3430**	0.037
Ecotype* Seasonality* Distance	2	**12.3303**	<0.0001	**14.6348**	<0.0001	**4.4605**	0.013	**13.6231**	<0.0001

N = 229. A * indicates interaction terms.

Seasonality effects on biomass, nitrogen, ADF and food quality are different for the two ecotypes. Effects of distance class are the same for the alpine region and mountain steppe (check interaction terms in Table 2).

### Seasonality

Seasonality shows significant effects on nitrogen, ADF and food quality (Table 2). Effects depend on spatial scale (Table 3). Effects on biomass are more pronounced for close than for distant sites. Nitrogen concentration and quality decrease from July to September in the alpine region but show less differences in mountain steppe. For ADF, distant sites seem more affected by seasonal changes than close sites: significant changes are more pronounced for both distant sites than for the close site in the mountain steppe. At the close site in the alpine region, ADF does not change significantly.

**Table 3:** Single factor analyses for the main factors ecotype, seasonality and distance class in the alpine meadow (alpine) and mountain steppe (steppe).

Factor	Spatial scale	N	df	Biomass		Nitrogen		ADF		Quality	
				F	p	F	p	F	p	F	p
Season.	alpine dis.	58	2	3.0859	0.053	**47.15**	<0.0001	**11.776**	<0.0001	**40.202**	<0.0001
	alpine cl.	59		**9.4752**	<0.001	**22.226**	<0.0001	0.6739	0.514	**9.2926**	<0.001
	steppe dis.	57		3.3982	0.041	2.8519	0.07	**10.607**	<0.001	0.6073	0.549
	steppe cl.	55		**12.665**	<0.0001	**3.603**	<0.05	**5.31**	<0.01	**6.2688**	<0.01
Distance	alpine	117	1	**96.774**	<0.0001	**14.012**	<0.001	0.0952	0.758	**9.4725**	<0.01
	steppe	112		**68.904**	<0.0001	**20.016**	<0.0001	0.0362	0.8495	**8.7536**	<0.01

### Distance from a river

Biomass, nitrogen and quality differ between distant and close sites in both alpine region and mountain steppe (Table 3, Figure 4).

Biomass is higher at distant sites while N and quality is higher in the vicinity to temporary human settlements. Interaction of distance class and seasonality is significant for biomass, ADF and quality in the alpine region and the mountain steppe.

At close sites in the alpine meadow and mountain steppe as well as in all plots in the semi-desert there is a significant negative correlation of DOM and quality. At distant sites of the alpine meadow and mountain steppe there is no such correlation. For the Pearson correlations between biomass and quality, data of the semi-desert were included (Table 4).

**Table 4:** Pearson’s correlation analysis between food quality (measured as the ratio of nitrogen/ADF) and DOM for pooled data, single pooled ecotypes and each distance class per ecotype.

Data Set	N	r coefficient	p
alpine region, mountain steppe and semi-desert	286	−0.3115764	>0.0001***
alpine region	115	−0.2951415	>0.01**
mountain steppe	112	−0.3664732	>0.0001***
Semi-desert	59	−0.6854251	>0.0001***
alpine region, distant	59	−0.06731023	0.622
alpine region, close	56	−0.3052649	0.019*
mountain steppe, distant	57	−0.1258348	0.355
mountain steppe, close	55	−0.3886314	0.003**

***p≤0.0001,

**p≤0.01,

*p≤0.05.

## Discussion

In the Western Mongolian pasture systems food quantity and quality change in time and space. At all three ecotypes seasonal changes were found for nitrogen and food quality, while the distance to a temporary settlement affected both quantity and quality. Different ecotypes in Khovd Aimag provide fodder of different quality at the same time of year. The natural temporal dynamics of main pasture plants is well known, e.g. Tserendash and Erdenebaatar [32] describe an early summer flowering with maximal nitrogen contents of species of *Koeleria*, *Poa* and *Agropyron* at steppe ecosystems. At the same time of the year species of *Carex* in meadow ecosystems show late flowering. Plants serving as fodder during the growing season can show compensatory growth after grazing producing new material rich in nitrogen and poor in fiber [33]. As a consequence the quality of the pasture is improved. All study sites were used as pasture to some extent during the summer. Thus compensatory growth as suggested in literature [34]–[36] could be a possible reaction of plants to grazing. A study from a meadow steppe in Inner Mongolia within a similar plant community shows a clear increase in total nitrogen contents under increasing grazing intensity for e.g. *Koeleria cristata*, *Artemisia frigida* and species of *Stipa*, *Thalictrum*, and *Pulsatilla* [37]. Nitrogen contents in grasses and sedges were generally lower than of forbs, and leguminous plants showed highest nitrogen contents in the same study.

Negative correlations between biomass and the chemical quality of the vegetation as shown in Table 4 have been found in other studies on grazing and its impact on the vegetation [38]. The negative correlation was found only for close study sites but not for the distant sites. This could indicate more active plant production and new growth after grazing in these areas. In all ecotypes nitrogen contents were higher close to the river than at more distant sites.

However, this picture of compensatory growth should not be generalized. Whether plants can compensate or not and how they compensate can depend on the type of damage, abiotic conditions such as rainfall or nutrient availability, and the effect of nutrient influx caused by the feces of livestock itself [33], [38], [39]. In addition, plants seem to have the option of differential compensation within the same individual, resulting in plant material of diverging quality within the same plant [31].

At the studied sites nitrogen contents are significantly higher for soils in the alpine region than for soils of mountain steppe or semi-desert (analyzed soil parameter from data of [40]). In the alpine region the availability of nitrogen in the soil could facilitate compensatory growth, or allow different plant reactions than soil conditions in the mountain steppe or semi-desert.

The decreasing quality in alpine vegetation as a result of reduced nitrogen concentrations is likely due to the seasonal changes in weather. Temperature dropped and any precipitation resulted in snowfall. Many plants started to wither in late August, which follows the described decrease in nutritive value until October in the literature [32]. Compensatory growth under these conditions was unlikely.

Changing patterns in nitrogen contents during the season were very different in the three ecotypes. A clear decrease was found in alpine meadow, mostly at the distant site. In contrast, nitrogen concentrations increase in the semi-desert between July and September. One reason for the different dynamics is surely the different climatic conditions and the shift in season. Winter was starting in the alpine regions while summer had reached its peak in the semi-desert. While the vegetation in the alpine region had already dried up, the vegetation of the semi-desert had just started growing after heavy rainfalls in August. The vegetation of the mountain steppe showed no clear signs of change, neither in nitrogen contents nor in outer appearance. Thus, seasonality in the three ecotypes cannot be defined through the Julian calendar but should be defined phenologically or climatically. Given the successive changes over the growing season found in this and many other studies, the decline of nitrogen content in semi-desert and mountain steppe is to be expected later during the year, with the change of climate during autumn and winter [17], [21], [32], [41]. Cattle and sheep are likely to avoid food with nitrogen content below 1.12%; dairy cows prefer food with nitrogen contents above 1.9% [42]. Nitrogen contents in the alpine region are about 1.2% in August and September. Thus, leaving this region in August is advantageous from a biochemical point of view.

The distance to temporary human settlements seems to be an important factor for the nitrogen and even more for DOM in the alpine region and the mountain steppe. Although vegetation cover is lower in semi-desert and mountain steppe than in alpine region, differences in DOM are much clearer between distance classes than between ecotypes. This might be due to the taller habit of grass communities that compensate sparse cover as far as total biomass is concerned.

Reduced biomass as a consequence of grazing is often found, but intermediate grazing can increase plant production [42]–[44]. Thus, the differences in biomass found between the two distance classes could be due to reduced growth at close sites or increased plant production at distant sites or even both. The study in Inner Mongolia [37] reveals consistent decrease of plant biomass with increasing grazing intensity for species of *Stipa*, *Bupleurum* and *Pulsatilla* and increasing biomass for *Artemisia frigida*. This can only be revealed by exclosure experiments or longterm data.

The present study did not indicate negative effects of grazing such as a shift in species composition, decrease in fodder quality due to damage or a shift to unpalatable species or intensive erosion. Only very few species showed a significantly different coverage in close and distant plots: In the alpine meadow *Thalictrum alpinum* and *Oxytropis altaica* showed higher coverage in distant plots while *Androsace fedtschenkoi*, *Oxytopis oligantha*, *Artemisia argyrophylla, Stellaria pulvinata and Festuca ovina* showed significant higher cover in close plots [40]. In the steppe, *Koeleria cristata* and *Poa attenuata* showed higher coverage in distant plots whereas *Artemisa rutifolia* and *Convolvulus ammanni* showed higher coverage in close plots. Still, the significances were low and the community structure seemed not to be influenced heavily by the current pasture usage. In contrast to that, Lkhagva et al found up to 50% of the biomass consisting of unpalatable species in boreal steppe ecosystems under high grazing pressure and grazing related increase of grazing tolerant species was observed in steppe and riparian zones under high grazing pressure [27]. However, conclusions based on short-term studies have to be interpreted with care as pasture characteristics in dry ecosystems can be highly variable between years. Here, differences of more than 275% in biomass production are not uncommon between years, especially for arid and highly seasonal regions where the importance of abiotic factors can override the effect of grazing [45] and plant productivity is much more variable than in ecosystems with higher precipitation [46].

## Supporting Information

Table S1 Species List: List of species found in more than 5% of all plots in an ecotype.
(DOCX)

Code S1 R code for statistical analysis.
(R) Click here for additional data file.

Data S1 Data table used for ANOVA in R (semi-desert excluded).
(TXT)

Data S2 Data table used for Pearson’s correlation analysis (semi-desert included).
(TXT)
