# Supplementary material for: Pasture Characteristics in Three Different Ecotypes at Khovd Aimag, Western Mongolia
Source: PLoS One. 2014 Jul 24;9(7):e102892. doi: 10.1371/journal.pone.0102892 (PMC4109948; doi:10.1371/journal.pone.0102892)
Supplement: Table S1 — Species List: List of species found in more than 5% of all plots in an ecotype. (DOCX) [file pone.0102892.s001.docx]

Supplementary table: Species List

List of species found in more than 5% of all plots in an ecotype

| **Species** | **Alpine Region** | **Mountain Steppe** | **Semi-Desert** |
| --- | --- | --- | --- |
| *Agropyron cristatum*  *Agropyron nevskii*  *Amblynotus rupestris*  *Androsace bungeana*  *Androsace fedtschenkoi*  *Allium mongolicum*  *Allium tenuissimum*  *Anabasis brevifolia*  *Arenaria capillaris*  *Artemisia adamsii*  *Artemisia anethifolia*  *Artemisia argyrophylla*  *Artemisia caespitosa*  *Artemisia dolosa*  *Artemisia frigida*  *Artemisia laciniata*  *Artemisia macrocephala*  *Artemisia mongolica*  *Artemisia obtusiloba*  *Artemisia phaeolepis*  *Artemisia pycnorhiza*  *Artemisia rutifolia*  *Artemisia* sp.  *Asterothamnus heteropappoides*  *Astragalus brevifolius*  *Astragalus lupulinus*  *Astragalus vallestris*  *Atraphaxis bracteata*  *Atriplex* sp.  *Axyris prostrata*  *Bupleurum bicaule*  *Caragana bungei*  *Caragana leucophloea*  *Caragana pygmaea*  *Carex duriuscula*  *Carex melanocephala*  *Carex rupestris*  *Cerastium pusillum*  *Chamaerhodos altaica*  *Chamaerhodos erecta*  *Chamaerhodos sabulosa*  *Chamaerhodos* sp.  *Chenopodium acuminatum*  *Chenopodium album*  *Clausia aprica*  *Cleistogenes squarrosa*  *Convolvulus ammani*  *Dontostemon integrifolius*  *Dontostemon senilis*  *Draba fladnizensis*  *Draba lanceolata*  *Draba nemorosa*  *Dracocephalum foetidum*  *Dracocephalum fruticulosum*  *Dracocephalum moldavicum*  *Elymus secalinus*  *Ephedra sinica*  *Festuca lenensis*  *Festuca ovina*  *Gentiana pseudoaquatica*  *Gentiana* sp.1  *Gentiana* sp.2  *Gueldenstaedtia monophylla*  *Gypsophila desertorum*  *Heteropappus hispidus*  *Hymenolobus procumbens*  *Iris potaninii*  *Iris tenuifolia*  *Kobresia humilis*  *Kobresia smirnovii*  *Koeleria cristata*  *Krascheninnikovia ceratoides*  *Krylovia eremophila*  *Lagotis integrifolia*  *Lappula stricta*  *Leontopodium ochroleucum*  *Linaria altaica*  *Melandrium apetalum*  *Melandrium brachypetalum*  *Neopallasia pectinata*  *Orostachys spinosa*  *Oxytropis altaica*  *Oxytropis micrantha*  *Oxytropis myriophylla*  *Oxytropis oligantha*  *Oxytropis strobilacea*  *Oxytropis tenuis*  *Pachyneurum grandiflorum*  *Panzerina lanata*  *Papaver pseudocanescens*  *Pedicularis uliginosa*  *Pennisetum centrasiaticum*  *Peucedanum hystrix*  *Phlomis tuberosa*  *Plantago despressa*  *Plantago komarovii*  *Poa altaica*  *Poa attenuata*  *Poa tianschanica*  *Polygonum aviculare*  *Polygonum viviparum*  *Potentilla astragalifolia*  *Potentilla bifurca*  *Potentilla conferta*  *Potentilla mongolica*  *Potentilla sericea*  *Potentilla soongorica*  *Potentilla* sp.  *Primula farinosa*  *Ptilotrichum canescens*  *Pulsatilla bungeana*  *Ranunculus pedatifidus*  *Salsola collina*  *Saussurea leucophylla*  *Saussurea pricei*  *Saxifraga hirculus*  *Scorzonera ikonnikovii*  *Senecio campester*  *Sibbaldianthe adpressa*  *Silene altaica*  *Silene* sp*.*  *Smelowskia alba*  *Stellaria crassifolia*  *Stellaria dichotoma*  *Stellaria gypsophiloides*  *Stellaria pulvinata*  *Stipa glareosa*  *Stipa krylovii*  *Taraxacum brevirostre*  *Taraxacum dissectum*  *Thalictrum alpinum*  *Thalictrum foetidum*  *Tribulus terrestris*  *Trisetum litwinowii*  *Vicia cracca*  *Youngia tenuifolia* | +  +  +  +  +  +  +  +  +  +  +  +  +  +  +  +  +  +  +  +  +  +  +  +  +  +  +  +  +  +  +  +  +  +  +  +  +  +  +  +  +  +  +  +  +  +  +  +  +  +  +  +  +  + | +  +  +  +  +  +  +  +  +  +  +  +  +  +  +  +  +  +  +  +  +  +  +  +  +  +  +  +  +  +  +  +  +  +  +  +  +  +  +  +  +  +  +  +  +  +  +  +  +  +  +  +  +  +  +  +  +  +  +  +  +  +  +  +  +  +  +  +  + | +  +  +  +  +  +  +  +  +  +  +  +  +  +  +  +  +  +  +  +  +  +  +  +  +  +  +  +  +  +  +  +  +  +  +  + |
